# Successful intubation with a flexible optical stylet in a patient with predictors of difficult airway using pharyngeal clearance technique with a laryngoscope: A case report

**DOI:** 10.3892/mi.2025.239

**Published:** 2025-05-05

**Authors:** Alexis Israel Bonilla González, Jimmy Gabriel Rivas Alpuche, Hugo Enrique Navarrete García, Lobsang Harish Wong Salazar, Carlos Martín Atondo Laguna, Perla Alejandra Avilés Sánchez

**Affiliations:** 1 Department of Anesthesiology, High Specialty Medical Unit (UMAE), Mexican Institute of Social Security (IMSS), 97150 Mérida, Yucatán, Mexico; 2 Faculty of Medicine, Autonomous University of Yucatán (UADY), 97000 Mérida, Yucatán, Mexico

**Keywords:** intubation, difficult airway, flexible optical stylet, pharyngeal clearance technique

## Abstract

General anesthesia (GA) is associated with risks, including inadequate ventilation, which significantly contributes to morbidity and mortality. A difficult airway (DA) is characterized by the inability to achieve adequate ventilation and occurs in 1.2 to 3.8% of adults undergoing GA. Although laryngoscopy is the standard method for intubation, alternatives, such as video laryngoscopy and optical stylets (OS) are particularly valuable in DA cases. The present study describes the case of a 61-year-old female patient with a history of diabetes, hypertension and thyroid cancer who underwent surgery for tumor resection following cancer recurrence. Given the presence of a DA, intubation was successfully performed using a flexible OS with a pharyngeal clearance technique. Anesthesia was maintained with desflurane and fentanyl, and the procedure proceeded smoothly with minimal blood loss. The patient remained hemodynamically stable throughout the surgery and was extubated awake without complications. The present case report highlights the successful intubation of a patient with a predicted DA using an OS and a pharyngeal clearance technique. The OS provides several advantages, including reduced autonomic stimulation, portability and cost-effectiveness. However, further research on intubation times is required to refine this approach and facilitate its comparison with other methods in clinical practice.

## Introduction

General anesthesia (GA) poses significant risks, particularly related to inadequate ventilation (1,2), which accounts for 40% of anesthesia-related deaths (3). According to the Fourth National Audit Project (NAP4), intraoperative hypoxia is fatal in 12% of these cases, while an additional 9% of patients succumb post-operatively (4). Among the survivors, 25% experience severe complications, such as cerebral hypoxia, pulmonary edema, or cardiorespiratory arrest (5). Furthermore, the study by Cumberworth *et al* (6) reported a global complication rate of 0.028%, with an average of 46 intensive care unit admissions per million procedures. Several factors further exacerbate ventilation challenges during GA, including obesity, maxillary hypoplasia, macroglossia and cervical instability (7,8). Additionally, conditions such as cancer and hypotension contribute to increased peri-operative risks (9,10).

The American Society of Anesthesiologists (ASA) (2) defines a difficult airway (DA) as a situation in which an anesthesiologist is unable to provide adequate ventilation despite the proper use of techniques and ventilation devices (11,12). It is estimated that DA occurs in 1.2 to 3.8% of adults undergoing GA (13), with this prevalence rising to as high as 22% in certain populations (14-16). Additionally, NAP4 reports that mask ventilation fails in 1 out of 1,500 attempts, while endotracheal intubation is unsuccessful in 1 out of 2,000 procedures. The ‘cannot intubate, cannot ventilate’ scenario occurs in 1 out of every 5,000 patients, often with severe consequences (17).

Although predictive indexes for DA exist, some patients experience unexpected ventilation difficulties, necessitating intubation (18,19). Intubation involves inserting a tracheal tube through the mouth or nose and is commonly performed during GA (20). The standard protocol utilizes laryngoscopes with a Macintosh blade, achieving a success rate of 44 to 87% (21). However, alternative techniques have been developed for patients with DA (22), including video stylets and video laryngoscopes, which enable the continuous monitoring of the hypopharynx during tracheal tube insertion into the glottis (23).

Video laryngoscopy enhances visualization in patients with a DA and increases the success rate of first-attempt intubation (24), while also reducing the incidence of hypoxemia (20). Additionally, it is associated with a lower risk of sympathetic overstimulation and traumatic injury (23). However, the use of personal protective equipment during the COVID-19 pandemic posed challenges for intubation with this technique, prolonging the procedure and leading to the increased adoption of optical stylet (OS)-based techniques (25).

The OS is an instrument derived from lighted stylets, originally introduced by Berci and Katz (26) in 1979 for endotracheal intubation. This device ranges in length from 42 to 80 cm, with an average diameter of 5.5 to 10 mm. It consists of a long, slender body through which the endotracheal tube is inserted. Additionally, a small video camera positioned at the tip of the stylet captures and transmits images to an integrated monitor (27). The OS allows for intubation to be performed either by a single operator or with assistance (28). Due to its enhanced maneuverability, accessibility and lower cost, the OS is currently recommended for patients with a DA (29,30).

A wide variety of OS devices are currently available, differing in optical and morphological features, tip angulation and flexibility (31). Its clinical use has been validated through a well-documented case series, each incorporating slight modifications to the technique. Currently, several variations of the original approach of Dr Bonfils' exist, including those proposed by Halligan and Chartres (32), both of which have proven effective in patients with a DA. Despite the advantages of OS for intubation, its use remains limited in the current setting. Therefore, the present study describes the case of a patient with a predicted DA who was successfully intubated using an OS in combination with the pharyngeal clearance technique and a laryngoscope.

## Case report

The present study describes the case of a 61-year-old female patient who was treated at the High Specialty Medical Unit (UMAE), Mexican Institute of Social Security (IMSS), 97150 Mérida, Mexico with a history of type 2 diabetes mellitus and systemic arterial hypertension, for which she was undergoing treatment. She had been diagnosed with papillary thyroid carcinoma in 2018 and underwent a modified right neck dissection followed by adjuvant radiotherapy in 34 sessions. Since then, she had been on hormone replacement therapy with 150 µg oral levothyroxine per day. Additionally, she had undergone a bilateral modified radical axillary dissection in 2022 and a parotid tail resection in 2023, both without complications.

**Figure 1:**
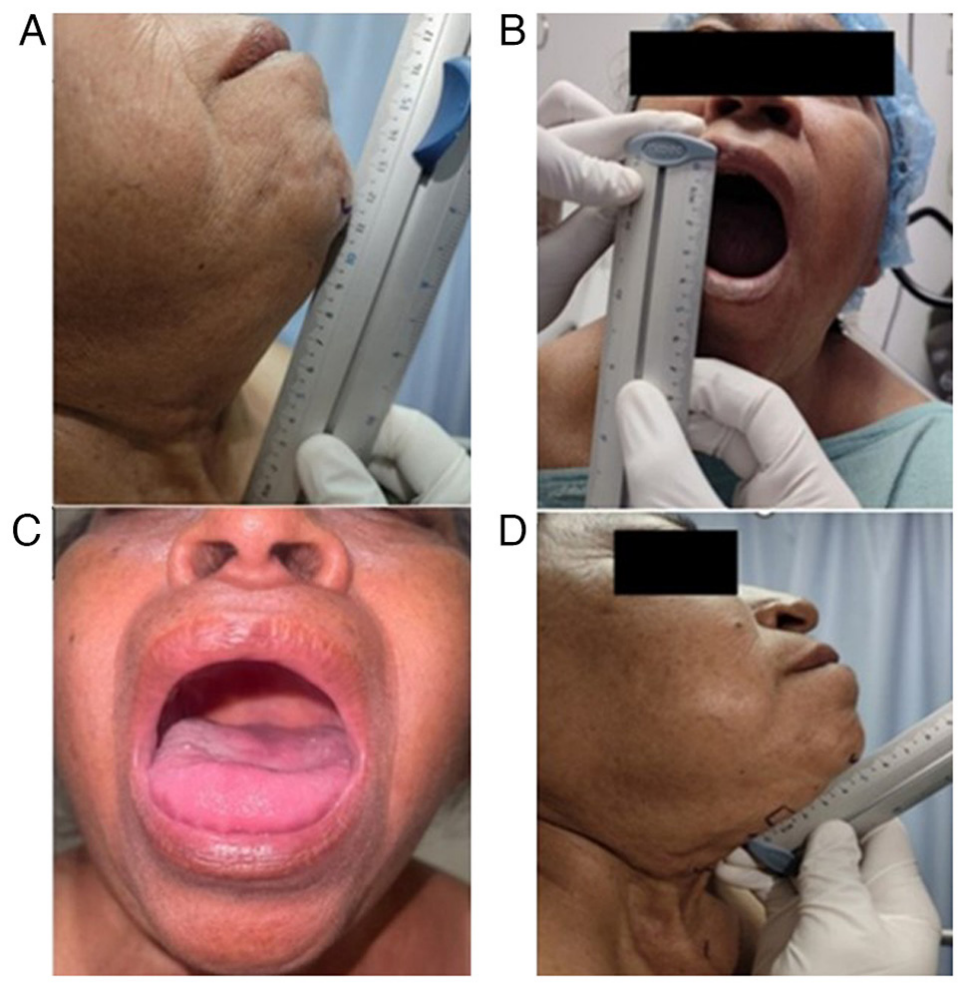
Pre-anesthetic airway assessment. (A) Sternomental distance grade III (11.5 cm). (B) Inter-incisal distance grade I (3.5 cm). (C) Mallampati III (marked restriction in tongue movement shown). (D) Patil Aldreti III (5.5 cm).

The patient was scheduled for a radical neck tumor resection due to the recurrence of papillary thyroid carcinoma. A physical examination revealed the following vital signs: Blood pressure, 177/79 mmHg; respiratory rate, 20 breaths/min; heart rate, 66 bpm; temperature, 37.1˚C, oxygen saturation (SpO_2_), 97%; body weight, 67 kg; height, 1.38 m; and body mass index, 35.2 kg/m². Airway assessment revealed incomplete dentition, a normal-sized tongue with restricted movement and protrusion, Mallampati III, Patil-Aldreti III (5.5 cm), sternomental distance III (11.5 cm), and interincisal distance I (3.5 cm) (Fig. 1). The Bellhouse- Doré classification was II (one-third mobility), and the Brodsky index was negative (34 cm).

**Figure 2:**
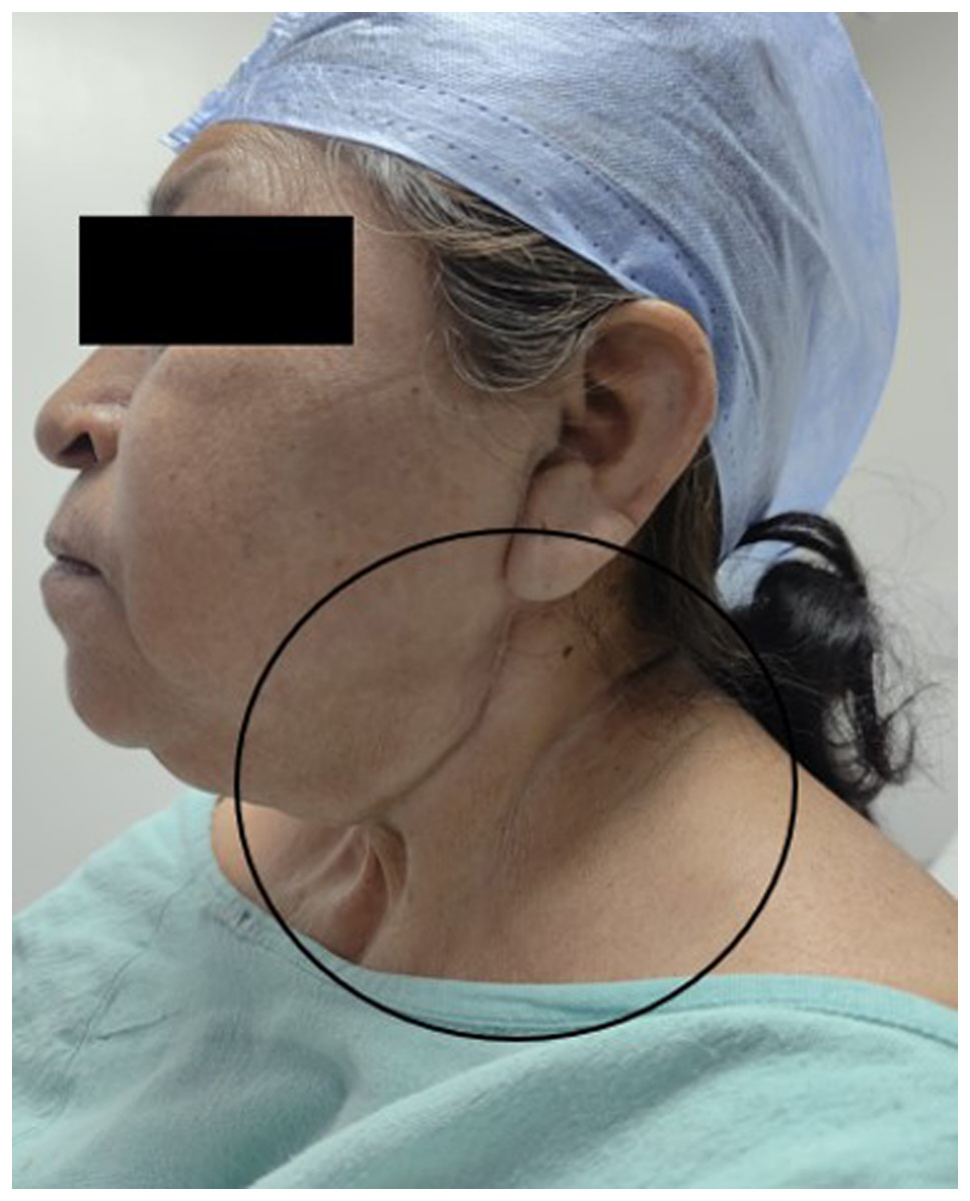
Image of the patient illustrating post-radiation cervical fibrosis. The neck region displays surgical scars and signs of post-radiation fibrosis (34 sessions of radiotherapy), which limit proper cervical extension.

**Figure 3:**
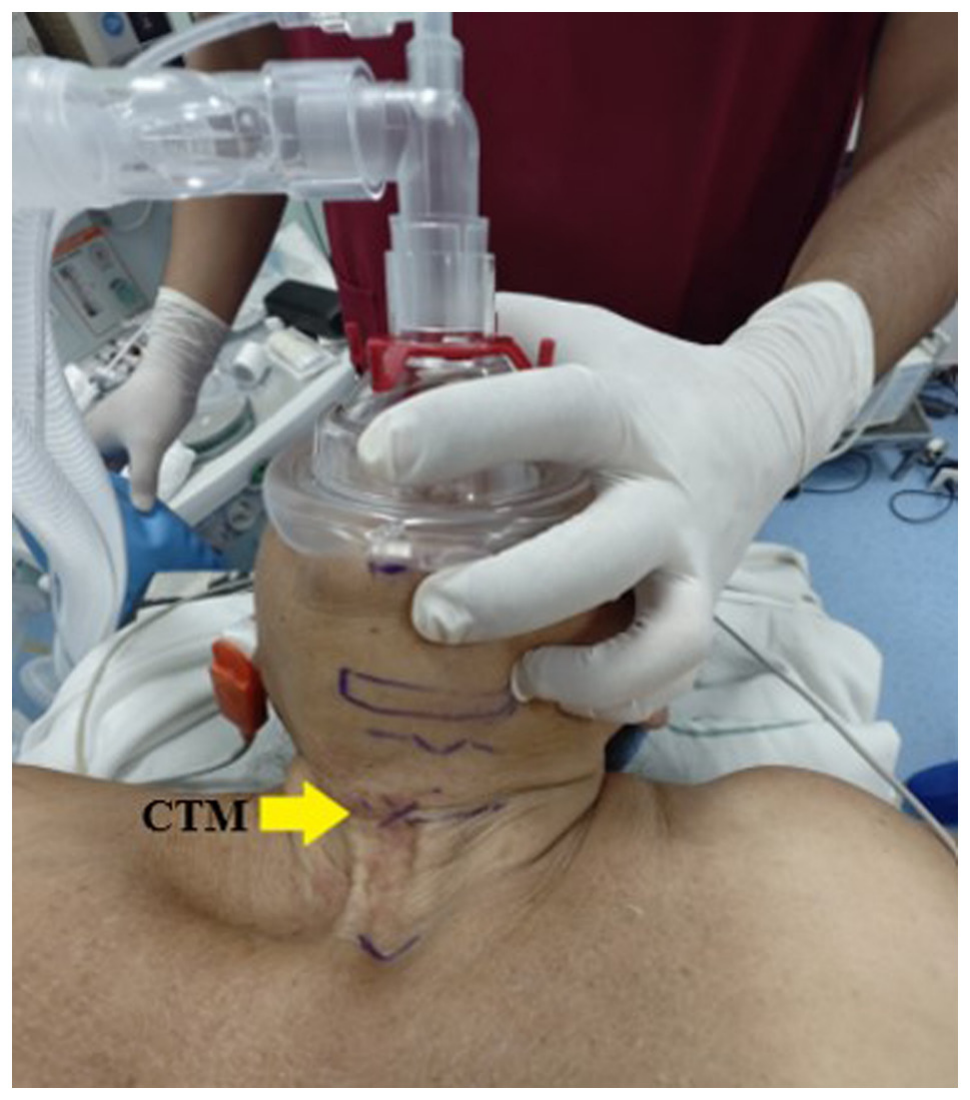
Marking of laryngeal structures. The areas marking the laryngeal structures in the neck are visible, with the cricothyroid membrane located off the midline. CTM, cricothyroid membrane.

Additionally, the neck exhibited surgical scars and signs of post-radiation fibrosis, which limited proper cervical extension (Fig. 2). Laryngeal structures were difficult to palpate, prompting an ultrasound examination to identify and mark the cricothyroid membrane with a clip using a linear probe in both longitudinal and transverse planes (Fig. 3).

Based on these findings, the surgical and anesthetic risk was classified as E3B, ASA III. A plan for balanced general anesthesia with invasive monitoring was established, incorporating airway management using a flexible intubation OS. Following thorough equipment and instrument check, the patient was transferred to the operating room, placed in the supine position and connected to continuous vital signs monitoring. Preoxygenation was then performed using a sealed facial mask with a tidal volume technique and 100% FiO_2_ for 3 min. For anxiolysis, 1.5 mg intravenous (IV) midazolam PiSA^®^ were administered, followed by 250 µg IV fentanyl PiSA^®^, 100 mg IV lidocaine PiSA^®^ and 35 mg IV rocuronium PiSA^®^.

**Figure 4:**
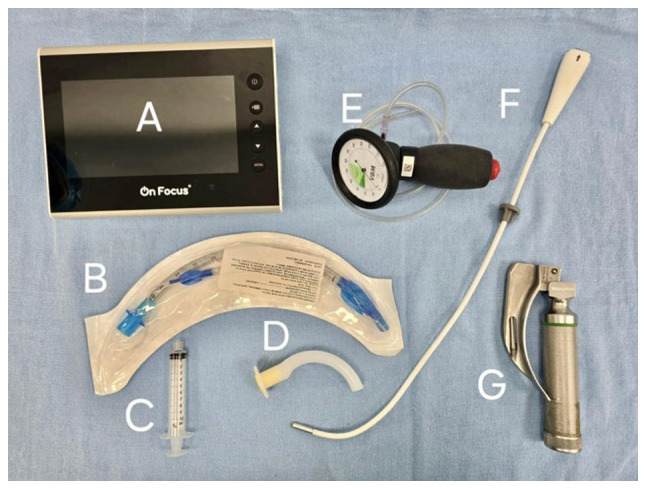
Illustration of the airway instrumentation materials used. (A) 7-inch HD LCD screen with a resolution of 1280x720 px. (B) Flexometallic endotracheal tube with cuff, 6.5 mm internal diameter. (C) 10 cc syringe for pneumo-tamponade. (D) Guedel 90 mm. (E) Manometer for measuring intratracheal pressure. (F) Flexible optical intubation stylet 5 mm in diameter. (G) Laryngoscope with Mac 3 blade.

**Figure 5:**
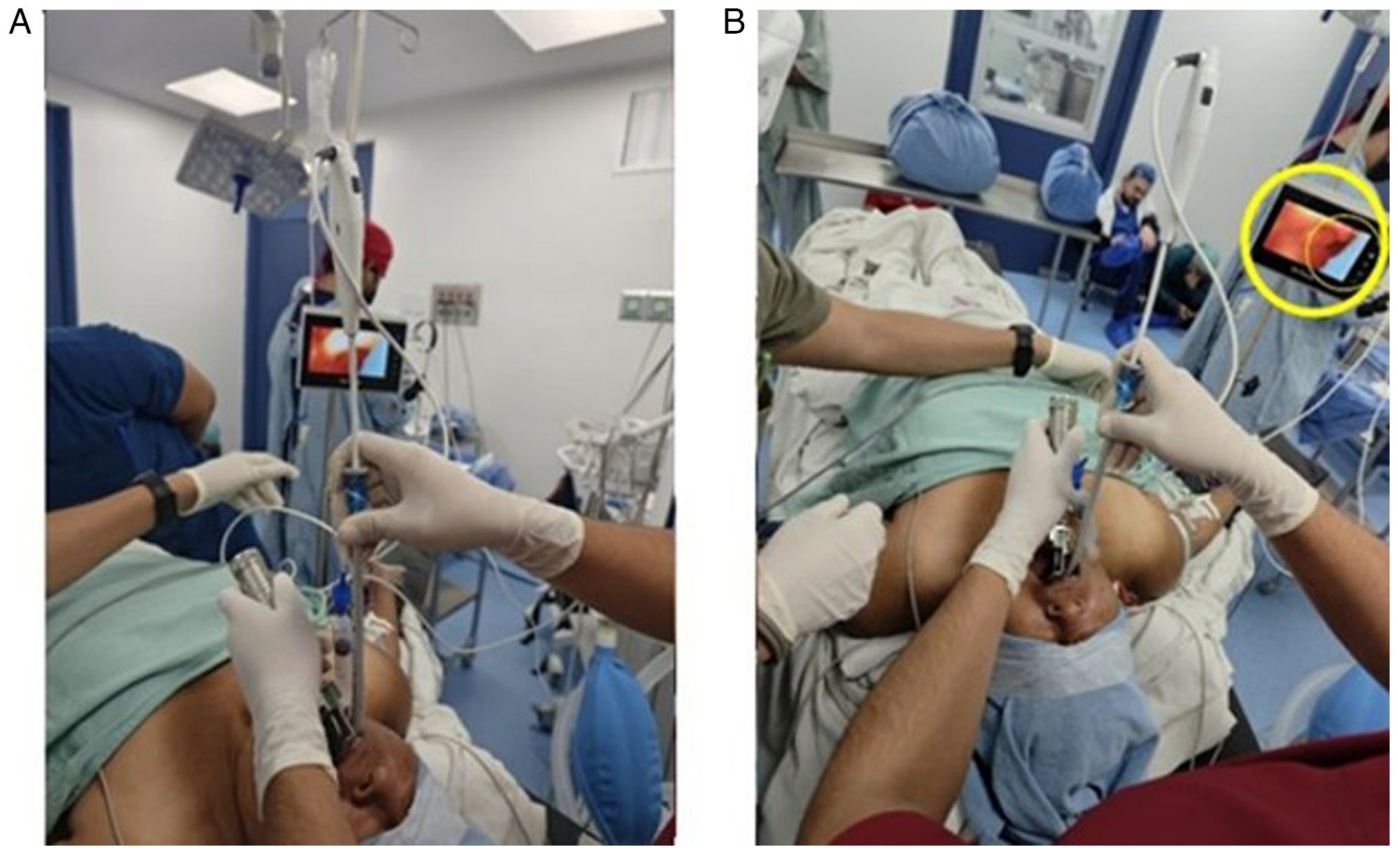
Pharyngeal clearance technique with laryngoscopy. (A) Intubation with a flexible optical stylet is observed, using the pharyngeal clearance technique with a Mac 3 laryngoscope blade, with a 6.5 mm internal diameter flexible metallic tube already rail guided. (B) Presence of the ‘Crescent Sign’ (circled) on the external screen of the device.

Anesthesia induction was achieved with 60 mg IV propofol (ALVARTIS PHARMA^®^). Upon obtaining entropy values of RE-49 and SE-45 with a train-of-four (TOF) ratio of 24%, the operator inserted a Mac 3 laryngoscope blade into the oral cavity using the non-dominant (left) hand to displace the tongue. With the dominant (right) hand, a flexible OS with a diameter of 5 mm, preloaded with a 6.5-mm internal diameter flexible metallic tube with a cuff, was introduced via the right retromolar approach (Fig. 4). An epiglottoscopy was performed, and once the glottis was visualized and identified, the tube was smoothly advanced into the trachea on the first attempt using the pharyngeal clearance technique with laryngoscopy. The procedure was atraumatic, and cuff inflation was achieved with a pressure of 30 cm H_2_O, confirmed by manometry (Fig. 5). The tube was then connected to mechanical ventilation, and proper positioning was verified through capnography and lung field auscultation

The surgical procedure was initiated with desflurane maintained at a minimum alveolar concentration of 0.8-0.9, an IV fentanyl PiSA^®^ infusion at 2-4 µg/kg/h (total, 300 µg) and a 2% IV lidocaine PiSA^®^ infusion at 1-2 mg/kg/h (total, 240 mg). Adjunct medications included 1 g IV paracetamol KENER^®^ and 2 g IV cefotaxime AMSA^®^.

Intraoperative blood loss was 30 ml, with a urine output of 1 ml/kg/h. Fluid management consisted of 1,000 ml Hartmann's solution and 500 ml 0.9% sodium chloride (NaCl) PiSA^®^, resulting in a total intake of 1,500 ml and a net positive balance of +140 ml. Following the procedure, the patient remained hemodynamically stable, with a mean arterial pressure of 75-80 mmHg, a heart rate of 65 bpm, a respiratory rate of 12 breaths/min, a temperature of 35.8˚C and an SpO_2_ of 100%. Secretions were gently aspirated.

Emergence was achieved through metabolic lysis, with effective spontaneous ventilation, the recovery of protective airway reflexes, entropy values of RE-93 and SE-92, a TOF ratio >90%, an appropriate response to verbal command, and spontaneous eye opening. The patient was extubated awake without complications.

The emergence was resolved through metabolic lysis, with effective spontaneous ventilation, recovery of protective airway reflexes, entropy values of RE-93 and SE-92, a TOF ratio >90%, an appropriate response to verbal commands, and spontaneous eye opening. The patient was extubated awake without complications.

## Discussion

The present study describes the case of a 61-year-old patient with predictors of a DA using a flexible OS and the pharyngeal clearance technique with laryngoscopy. The clinical characteristics of the patient reflect common sequelae of neck surgery and skin changes due to fibrosis secondary to radiotherapy, which restricts cervical extension. For these reasons, anesthetic protocols should always incorporate alternative strategies for cases in which traditional intubation techniques fail, with the OS being one of the recommended tools for patients with an anticipated DA (33).

The OS is one of several nonconventional intubation devices, along with blade laryngoscopes, optical bougies and stylets. These devices have been widely developed in recent years due to their ease of use and enhanced durability. They are available in rigid, semi-malleable and hybrid varieties, the latter being partially rigid and partially flexible (34). Initially, the most commonly used OS intubation technique was proposed by Dr Bonfils. In this method, the device was pre-treated with an anti-fog solution on the distal lens and preloaded with the endotracheal tube. It was then inserted through the retromolar region of the right cheek until reaching the posterior molars. At this point, the OS was redirected toward the midline to visualize the uvula, followed by the epiglottis and then the glottic opening, where the tube was smoothly advanced into the trachea (26).

Subsequently, Halligan and Charters (32) proposed several maneuvers to facilitate the use of the Bonfils OS. These maneuvers involved inserting the non-dominant hand into the mouth of the patient to apply forward traction on the mandible and, if necessary, on the tongue as well. This was followed by an external mandibular subluxation maneuver with maximal head extension. If these techniques failed to clear the airway, direct laryngoscopy was performed as a pharyngeal clearance strategy (32).

However, the integration of the OS into High Specialty Medical Unit (UMAE) s relatively recent, as direct and video laryngoscopy remain the standard alternatives for managing predicted DA. It is important to emphasize that, given the high prevalence of DA among in the patients treated at the UMAE (most of whom have oncological conditions) airway management plays a critical role in their prognosis and is a key aspect of our daily practice. To ensure proficiency with this technique, induction workshops using the OS were conducted with mannequins prior to its application in actual patients, allowing operators to improve their learning curve, as stated in previous research (35).

The OS is considered a non-conventional intubation method that, compared to direct laryngoscopy, induces less autonomic stimulation. Additionally, it provides several advantages, including greater portability, ease of disinfection, a short learning curve, lower cost and the ability to displace tumors in the oral cavity. These features contribute to its high first-attempt success rate (36), rendering it a safe and effective alternative comparable to ultrasound-guided techniques (37).

Moreover, recent studies have highlighted its superior utility over conventional methods. According to the study by Zhang *et al* (38), this technique enabled successful intubation in a patient who unexpectedly presented with a DA secondary to cervical hyperostosis. Similarly, Yang *et al* (39) described the case of a middle-aged male patient with multiple comorbidities who developed hypoxemia during an acute episode of COVID-19 and was successfully intubated and stabilized using this technique. Additionally, a series of cases involving patients with neck trauma who required cervical immobilization and encountered difficulties being intubated with a video laryngoscope were reported. As a result, the video stylet was used, and success in intubation was achieved due to its lower requirement for cervical manipulation (40).

It is important to highlight that the success of intubation reported herein could be further optimized by determining the average intubation time, allowing for an objective comparison with other methods. In this regard, Jhuang *et al* previously published a case series analyzing this parameter, reporting a duration range of 6 to 11 sec (41). Therefore, future studies evaluating the effectiveness of the OS are required to include the measurement of average intubation time, providing objective data that support the routine adoption of this instrument.

In conclusion, the integration of the OS into routine airway management for patients with a DA represents a promising strategy to improve clinical outcomes. As the prevalence of patients with comorbidities and prior surgical histories continues to increase, factors that significantly increase the complexity of ventilation during surgery, the OS stands out as an optimal alternative. Its adoption could enhance airway management protocols, contributing to safer and more effective anesthetic procedures.

## Data Availability

The data generated in the present study may be requested from the corresponding author.
